# The role of Aquaporins in tumorigenesis: implications for therapeutic development

**DOI:** 10.1186/s12964-023-01459-9

**Published:** 2024-02-09

**Authors:** Arkadyuti Bhattacharjee, Ankit Jana, Swagato Bhattacharjee, Sankalan Mitra, Swagata De, Badrah S. Alghamdi, Mohammad Zubair Alam, Ahmad Bakur Mahmoud, Zainab Al Shareef, Wael M. Abdel-Rahman, Chan Woon-Khiong, Athanasios Alexiou, Marios Papadakis, Ghulam Md Ashraf

**Affiliations:** 1 Morningside Graduate School of Biomedical Sciences, University of Massachusetts Medical School, Worcester, USA; 2 Department of Biological Sciences, National University of Singapore, Singapore, 117558 Singapore; 3 KoshKey Sciences Pvt Ltd, Canara Bank Layout, Karnataka, Bengaluru, Rajiv Gandhi Nagar, Kodigehalli 560065 India; 4 KIIT School of Biotechnology, Kalinga Institute of Industrial Technology (KIIT-DU), Bhubaneswar, Odisha India; 5 Department of English, DDE Unit, The University of Burdwan, Golapbag, Burdwan, West Bengal 713104 India; 6 Department of Physiology, Neuroscience Unit, Faculty of Medicine, King Abdulaziz University, Jeddah, Saudi Arabia; 7 Pre-clinical Research Unit, King Fahd Medical Research Center, King Abdulaziz University, Jeddah, Saudi Arabia; 8 Department of Medical Laboratory Sciences, Faculty of Applied Medical Sciences, King Abdulaziz University, Jeddah, Saudi Arabia; 9 College of Applied Medical Sciences, Taibah University, Almadinah, Almunwarah 71491 Saudi Arabia; 10 College of Medicine, and Research Institute for Medical and Health Sciences, Department of Basic Medical Sciences, University of Sharjah, Sharjah, 27272 United Arab Emirates; 11 College of Health Sciences, and Research Institute for Medical and Health Sciences, Department of Medical Laboratory Sciences, University of Sharjah, Sharjah, 27272 United Arab Emirates; 12 University Centre for Research & Development, Chandigarh University, Chandigarh-Ludhiana Highway, Mohali, Punjab India; 13 Department of Research & Development, Funogen, Athens, Greece; 14 Department of Research & Development, AFNP Med, 1030 Wien, Austria; 15 Department of Science and Engineering, Novel Global Community Educational Foundation, Hebersham, NSW 2770 Australia; 16 Department of Surgery II, University Hospital Witten-Herdecke, Heusnerstrasse 40, University of Witten-Herdecke, 42283 Wuppertal, Germany

**Keywords:** Aquaporins, Cancer, Tumours, Water channel, Carcinoma, Therapeutic target

## Abstract

Aquaporins (AQPs) are ubiquitous channel proteins that play a critical role in the homeostasis of the cellular environment by allowing the transit of water, chemicals, and ions. They can be found in many different types of cells and organs, including the lungs, eyes, brain, glands, and blood vessels. By controlling the osmotic water flux in processes like cell growth, energy metabolism, migration, adhesion, and proliferation, AQPs are capable of exerting their regulatory influence over a wide range of cellular processes. Tumour cells of varying sources express AQPs significantly, especially in malignant tumours with a high propensity for metastasis. New insights into the roles of AQPs in cell migration and proliferation reinforce the notion that AQPs are crucial players in tumour biology. AQPs have recently been shown to be a powerful tool in the fight against pathogenic antibodies and metastatic cell migration, despite the fact that the molecular processes of aquaporins in pathology are not entirely established. In this review, we shall discuss the several ways in which AQPs are expressed in the body, the unique roles they play in tumorigenesis, and the novel therapeutic approaches that could be adopted to treat carcinoma.

## Introduction

Aquaporins (AQPs) are channel proteins from the major intrinsic protein (MIP) family that are present in nearly all living organisms [1]. AQPs play a crucial role in maintaining the cellular environment by facilitating transport of water, molecules, and ions. Aquaporins are present in various tissues throughout the body, including kidneys, lung airways, eyes, brain, glands, and vascular systems [2]. So far, 13 of the 17 recognized aquaporins have been identified in higher animals, including humans [3]. The 13 (AQP 0–12) human AQPs are classified into two major categories based on their permeability properties [4]. The first category of aquaporins (AQP 0, 1, 2, 4, 5, 6 & 8), though primarily viewed as water selective channels, are proven to have permeability to gases, urea, ammonia, hydrogen-peroxide, and ions [5, 6]. The next category, referred to as aquaglyceroporins (AQP 3, 7, 9 & 10), is permeable to glycerol, small solutes, and water [7]. Super-aquaporins (AQP 11 & 12), distinguished for their subcellular localization and atypical permeability properties, are less homologous with other aquaporins [8, 9]. However, the full range of solutes and ions that can be transported through AQPs are yet to be identified. Altered aquaporin functions have been associated with a surprising array of human diseases [10]. The substrate permeability and varying localization enable AQPs to regulate various functions, including osmotic water flux in cellular expansion, energy metabolism, migration, adhesion, and proliferation. Malignant tumour cells spreading through irregular aggression and metastasis within various parts of the body go through the process of cell metabolism, which is largely dependent on fluid in- and outflux mediated by aquaporins. Moreover, Dys-, up-, or down-regulation of aquaporin expression has been identified in more than 20 human cancer types [11]. AQPs, thus, assist in the angiogenic process, which plays a key role in the transportation of nutrients, reactive oxygen species, and other resources, thereby augmenting cancer progression [12–17] Over 20 different forms of human cancer have been shown to express AQPs, and there is a strong relationship between their expression and the prognosis of cancer patients. Globally, the incidence and mortality of cancer are rising quickly. In the twenty-first century, cancer is predicted to be the main cause of mortality worldwide and the single biggest obstacle to raising life expectancy [18]. Mainstream therapy, which aims primarily at inhibiting cancer proliferation, often causes prolonged and damaging side effects, encouraging a growing demand for new therapies targeting cancer metastasis [19, 20].

Researchers have proposed AQPs as exciting diagnostic biomarkers in cancer, oedema, and neuropathological diseases. In addition, the manipulation of ubiquitous presence and broad subtype-specific tissue expression of AQPs could serve as a beneficial anticancer therapeutic [21]. Although the molecular mechanisms of aquaporins in pathology are not fully recognized, recent studies clearly suggest that with the right combination of strategies, AQPs can serve as an effective blocking tool of pathogenic antibodies and metastatic cell movement [22, 23]. In this comprehensive review we aim at covering AQPs subtype-specific expressions in tissue, AQPs functions and specific roles in cancer progression, and AQPs potential as novel anticancer therapeutics.

## Aquaporins as a therapeutic target in Cancer

The prevalence of carcinoma in the elderly population is rising as the average human lifetime lengthens, with numbers anticipated to climb by 70% over the next two decades [18]. The passage of water particles through the cellular membrane is intimately tied to tumour metabolism, and AQPs are the primary transporters of water into and out of cells. Different levels of AQP expression have been linked to various types of metastasis in cancer, angiogenesis, and dynamic cellular changes. AQPs also enable and augment the transportation of reactive oxygen species (ROS), hence exacerbating carcinogenesis and tumour growth. Systemic therapy like chemotherapy, hormone therapy, and biological cure have become the typical treatment of various forms of carcinoma and are used in adjuvant, neoadjuvant, or palliative situations. In addition, toxic effect linked with presently accessible systemic therapies is widespread, often conflicting with the delivery of medication. In Table 1, we briefly presented different types of AQPs with their targeting pathways in specific carcinomas.

**Table 1:** Types of AQPs with their targeting pathways in specific carcinomas

AQPs	Chromosomal Location	Type of Carcinoma	Targeting Pathway/Molecules/Processes	Reference
AQP1	7p14	Astrocytoma, Ovarian, Colorectal, Hepatocellular, Breast, Lung, Endometrial, Adenoid cystic, Osteosarcoma	HIF-1a, Transcription of E box containing genes (by C-Myc), Promoter hypermethylation, TGF- β, H+ transport,	[14, 24–32]
AQP2	12q13	Endometrial, Lung	F-actin, Annexin-2 and Oestrogen receptor	[33, 34]
AQP3	9p13	lung cancer, colon cancer, cutaneous, renal, oesophageal and oral squamous cell carcinoma, Bladder, Oral squamous, NSCLC, Breast ductal	AKT-MMP pathway, PI3K-AKT-SNAIL, EGFR-ERK, CXCL12 signalling,	[35–41]
AQP4	18q22	Astrocytoma, Glioma	Protein Kinase C	[42, 43]
AQP5	12q13	Breast, Cervical, Endometrial, Prostate, Tongue squamous cell carcinoma, Lung, Colorectal, Gastric	RAS pathway, EGFR/ERK/p38 MAPK, p-SMAD2/3 pathway, NF-кB pathway.	[44–52]
AQP7	9p13	Thyroid	EGFR/ERK1/2	[53]
AQP8	16p12	Brain, Ovarian, Cervical, Acute leukaemia	EGFR/ERK1/2	[53, 54]
AQP9	15q22	Glioblastoma, Lung, Oesophageal	EGFR/ERK1/2	[55–57]

### AQP1

Aquaporin 1 (AQP1) is a channel-forming integral membrane protein of 28 kDa, initially identified in mammalian red blood cells and renal tubules (CHIP28) [58]. Aquaporin 1 (AQP1) is a tiny integral hydrophobic transmembrane protein whose primary function is trans-cellular water transport. Latest research has linked AQP1 upregulation to several cancer types as an independent predictive marker. This has motivated scientists to investigate the relationship between AQP1 and the biological processes of cancer.

AQP1 induces osmotic fluid transport throughout the cell membrane in reaction to an osmotic gradient formed by actin depolymerization and active influx of solute at the pioneering tip of moving cells, according to the findings of an investigation [59]. Kourghi et al. demonstrated that multiple AQP1 ion channel inhibitors that had no impact on AQP function hindered HT29 cell migration, with the extent of inhibition varying according to the effectiveness of the AQP1 ion channel blocker [60]. Simultaneously, Clapp and Escalera hypothesized that increased vascular porosity, that improves intracellular transport of water, can initiate an angiogenic cascades by increasing the extravasation of plasma proteins, which act as scaffolds for migratory endothelial cells [61]. Increased expression of AQP1 was seen in the Virchow–Robin region of astrocytoma wherein cancer cell penetration occurs, but expression is sparse in the necrotic core, proposing an association between AQP1 and tumour angiogenesis [24]. In the later phases of ovarian malignancies, the expression of the AQP1 protein was elevated, while cancer subgroups have varying degrees of either favourable or detrimental correlations with patient survival [25, 26]. Due to its association with tumour invasiveness and the fact that it is consistently overexpressed from the earliest to the latter stages of colorectal carcinogenesis, AQP1 is now considered a poor predictive biomarker of patient survival [27, 28]. Moon and colleagues reported that AQP1 expression in colonic adenoma, primary and secondary colon carcinoma, but not in healthy colonic mucosa, is involved in the earliest stages of carcinogenesis [62]. In another investigation, Jiang et al. found that alterations in osmotic water penetrability due to either extensive or inadequate expression of AQP1 channels affected the capacity of HT20 human colon cancer cells both in vitro and in vivo [63]. In cutaneous melanoma, AQP1 expression has been linked to a poor prognosis [64]. AQP1 overexpression was also identified in cholangiocarcinomas (CCs) and micro-vessels of hepatocellular carcinomas (HCCs) [65].

**Fig. 1:**
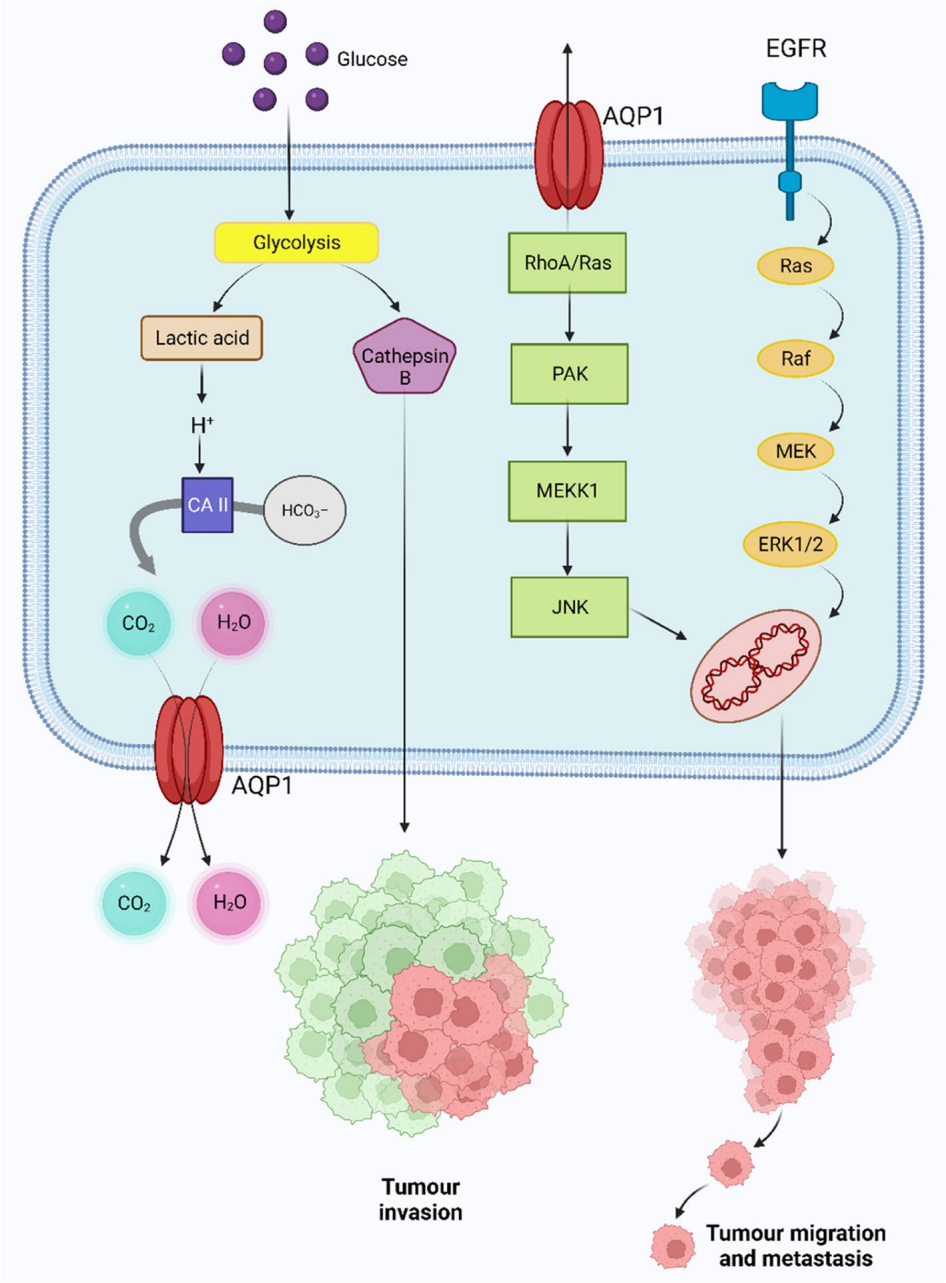
Role of AQP1 in Cancer Progression. Left- Putative mechanism of hypoxia-facilitated AQP1 expression in tumour cells. Hayashi and colleagues postulated that hypoxia facilitates AQP1 expression through glycolysis [87]. Lactic acid is produced during glycolysis, which also boosts the transcription of AQP1 and cathepsin B via E-box/ChoRE.Cathepsin Bcontributes to tumor invasion. An excess of H^+^ and intracellular acidosis are brought on by the increased lactic acid. Intracellular carbonic anhydrases (CA) catalyze the process that changes the excess H^+^ into H_2_O and CO_2_ from the interaction with HCO3^-^. While CO2 may leave the cell through the central hole of the AQP1 tetramer or diffuse across the plasma membrane, extra H_2_O created leaves the tumor cell through the water pores of the up-regulated AQP1 to prevent cytotoxic oedema. Right- Proposed mechanisms how AQP1 is involved with different signaling pathways to enhance tumour cell invasion and metastasis

The progression of breast and lung cancer was studied in mice lacking AQP1; in contrast to animals with normal AQP1 expression, tumour mass, vascular integrity, and lung invasion were reduced [66]. It was also revealed that cancer cells that overexpressed AQP1 were far more capable of cell motility, invasion, and metastasis. Hu and Verkman showed that the AQP1 expression at the advancing end of migratory cells increased the motility of breast cancer 4 T1 and mouse carcinoma B16F10 cell lines in vitro [14]. In breast cancer tissues, Yin et al. established a correlation between AQP1 and HIF1 expression [67]. Oestrogen-mediated AQP1 overexpression in breast cancer is downregulated by microRNA-320, was linked to a poor prognosis for patients with breast cancer [67–70]. The worst forms of basal-like breast carcinomas were found to have abnormally high amounts of the AQP1 protein [71]. Cell proliferation was increased in vitro when AQP1 was transfected into lung cancer cells [72]. In a separate study, it was revealed that AQP1 upregulation in the capillary endothelium of lung-carcinoma and mesothelioma tumours encouraged angiogenesis, hence promoting the development and propagation of cancer [29]. Hoque and colleagues used immunohistochemistry to examine the distribution of AQP1 in different types of primary cancer and found that it was increased in 62% of adenocarcinoma and 75% of bronchoalveolar carcinoma [72]. AQP1 promotes angiogenesis in lung cancer, and elevated levels of AQP1 expression were related with significant postoperative metastases and poor disease-free survival rates [35]. An investigation revealed that the subcellular location of increased AQP1 expression in the vascular endothelium of lung cancer capillary endothelial cells were accountable for angiogenesis [29, 73]. Moreover, Yang and colleagues reported that the epithelial ovarian tumours and AQP1 expression was associated with intra-tumoral microvascular density and the phase of the tumour [74]. Glioma invasiveness has also been linked with AQP1 overexpression [75]. In an experiment, Galan-Cobo and colleagues demonstrated that the cellular morphology alteration mediated by increased expression of AQP1 promotes tumorigenic advancement through the cell cycle and impedes the induction of apoptosis, as evidenced by greater cell size and intracellular ambiguity in contrast to the wild-type control [76]. C-Myc is frequently overexpressed in tumour cells and may effectively induce the expression of E-Box comprising genes, hence elevating AQP1 expression [77]. However, AQP1 promoter hypermethylation has been reported to be widespread in adenoid cystic carcinoma, with the overexpression of AQP1. Although the level of expression is unrelated to the outcomes and can be utilised as a prognostic marker [30]. Furthermore, hypoxia has also been found to facilitate tumour angiogenesis. In an experiment using the murine endothelial cell line EOMA, which over-regulates mutant versions of HIF-1 resilient to disintegration, it was shown that HIF-1 directly contributes to the hypoxic over-expression of the AQP1 promoter [78]. Researchers have speculated that the role of AQP1 in stabilizing the cadherin/ Lin-7/−catenin /F-actin complex enhances the invasive and migratory potential of cancer cells [79]. MMPs are often upregulated in several tumour cells and have been reported to accelerate tumour cell motility in vitro and metastasis in vivo. Researchers revealed that AQP1 siRNA inhibited MMP2 and MMP9 expression in LTEP-A2 and LLC lung cancer cell lines [80]. Another study found that as astrocytoma progressed from low to high grade, the intensity of AQP1 expression increased significantly [24]. Previously, Jiang et al. speculated that AQP1 is associated with RhoA and Rac after observing a significant upregulation of these tiny G proteins in moving HT20 colon carcinoma cells over-expressing AQP1, as well as an increase in the frequency of polarized actin production at the cells’ leading edges. RhoA expression was similarly reduced in two osteosarcoma cell lines, U2OS and MG63, following AQP1 down-regulation by shRNA, which was associated by a decrease in proliferation [31]. Interestingly, the mechanisms of AQP1-induced cell migration and metastasis in colon cancer cells were found to be associated with actin protein re-localization and RhoA and Rac activation [27]. Wu et al’s GSEA (Gene set enrichment study) revealed a significant link between AQP1 up-regulation and the TGF- signaling pathway in osteosarcoma, and the production of TGF-β1 and TGF-β2 was decreased in U2OP and MG63 cell lines when AQP1 expression was lowered by shRNA [31].. AQP1 may be implicated in lung cancer cell invasion and migration, which AQP1-shRNA able to inhibit [73]. An experiment involved intertumoral injections of AQP1 siRNAs used in RNA interference research in murine models. After 6 days of therapy, tumours treated with AQP1 siRNA exhibited a 75% decrease in volume in comparison to controls which were related to a substantial drop in expression of the endothelium marker factor VIII. The incidence of lung metastases was also increased, and the osmotic water permeability of the plasma membrane was increased by a factor of 5–10 in B16F10 melanoma cells and 4 T1 mammary gland tumour cells after intravenous infusion of tumour cells exhibiting AQP1 expression [14]. Xiang et al. suggested that carbonic anhydrase inhibitors’ suppressive activity on AQP1 might provide a repressive consequence on cancer invasion and upregulation [81]. Several scientists studied different aspects of malignant pleural mesothelioma – Angelico et al. conducted experiments on immunohistochemical expression of AQP1 in Fluoro-Edenite-induced malignant mesothelioma and observed that AQP upregulation was linked to an enhanced median overall survival [82]. Jagirdar and colleagues also discovered that AQP1 inhibition lowered cell adhesion [83]. In another study, regardless of therapy, Kao et al. observed that AQP1 level was linked with outcome in malignant mesothelioma [84]. In squamous cell carcinoma (SCC), Lehnerdt and colleagues demonstrated that AQP1 is a biomarker for an aggressive subset of basaloid-like SCC, while Yamazato et al. showed that AQP1 expression is linked to a low prognosis [85, 86]. AQP1 has the ability to work with carbonic anhydrases to block hydrogen ions (H^+^) from entering the cell, resulting in enhanced lactic acid generation, preventing tumour cytotoxic oedema and reduces the pH of the extracellular space. The acidic extracellular environment induced glioma cells to produce cathepsin B, which boosted glioma cell invasion [87]. It is hypothesised that matrix metalloproteinases (MMPs) and the plasminogen activator cascade, of which cathepsin B is a part, contribute to enhanced movement, infiltration, and angiogenesis. Tumour cell apoptosis may be dysregulated due to cathepsin B’s ability to annihilate anti-apoptotic proteins such Bcl-xl, Bcl-2, and Bak (Fig. 1) [88]. Previous studies have shown that cathepsin B, along with AQP1 and LDH, is overexpressed in 9 L cells in a glycolytic environment [87]. A study showed that AQP1 silencing by siRNA in HMEC-1 human endothelium cells and WM115 human melanoma cells led to a scarcity of F-actin polarization at the plasma membrane and an inability to form a cord-like cluster in vitro [79].. Inhibition of AQP1 by AqB050 or siRNA knockdown resulted in a decreased cell growth in primary malignant mesothelioma cells obtained from pleural effusions [89].

In a mouse model of melanoma, Nicchia et al. reported that inhibiting AQP1-dependent angiogenesis restricts tumour development [90]. They performed RNA interference knockdown employing AQP1 siRNA on mice implanted subcutaneously with B16F10 murine melanoma cells, revealing aberrant tumour microvascular architecture with lower density. AQP1 molecular knockdown and inhibition dramatically reduced colon cancer cell migration, indicating that AQP1 is a promising target for the treatment of colon cancer. Acetazolamide, a carbonic anhydrase inhibitor, was discovered to suppress AQP1 expression, which shielded tumours from cytotoxic oedema by maintaining extracellular acidity and promoted tumour spread in glioma [91]. Rg3 inhibited migration of PC-3 M cells, an extremely metastatic prostate cancer cell line, via inhibiting AQP1 expression via the p38 mitogen-activated protein kinases cascade and transcription factors functioning on the AQP1 promoter [92]. By modulating AQP-1 water channel activity and protein expression in the Lewis lung cancer model, acetazolamide is eventually beneficial as an angiogenesis antagonist [81]. It may also reduce the formation of colon cancer xenograft tumours in mutant mice by reducing the production of the AQP-1 gene [93]. Tetraethyl ammonium (TEA) was revealed to be a reversible antagonist of AQP168 and was subsequently shown to be an inhibitor of AQP1, AQP2, and AQP4 [94]. Zhang et al. studied the effect of AQP1 inhibition on chemotherapy sensitivity of J82 human bladder cancer cells and reported that combination of AQP1 inhibition alongside MMC treatment could be a potential cure [95]. Imaizumi et al. researched the association between AQP1 expression and response to adjuvant chemotherapy in stage II and III colorectal cancer. Using immunohistochemistry (IHC) technology, they determined that AQP1 expression is a marker predicting a response to adjuvant chemotherapy [96].

### AQP 2

The ability of cells to keep their water balance is essential to their health and survival. The collecting ducts, the terminal part of a nephron, are responsible for reabsorbing water [97]. Aquaporin-2 (AQP 2), the water channel sensitive to vasopressin, plays a crucial role in regulating this process [98–100]. Under healthy hydrating conditions, AQP 2 is abundant in the collecting duct and is mostly stored in intracellular repositories of collecting duct cells. The hormone vasopressin is released from the posterior pituitary when the body is dehydrated. Water reabsorption from the urinary tubule is promoted when circulating vasopressin binds to the vasopressin V2 receptor on the basolateral membrane of the principal cells of the renal collecting duct. This triggers signal transductions that encourage AQP 2 movement from intracellular vesicles to the apical membrane.

The AQP 2-expression was shown to be elevated in endometrial tissues from individuals with endometrial carcinoma (EC) and endometriosis [33]. By modulating the expression levels of F-actin and annexin 2, AQP2 facilitates estradiol (E2)-induced migration, invasion, and adhesion. Furthermore, AQP 2 has also been proven to be linked to chemotherapy response in lung cancer patients [34]. Nevertheless, Estradiol (E2) dose-dependently elevated AQP 2 expression and dramatically enhanced migration, invasion, adhesion, and proliferation of Ishikawa cells in human EC, which was inhibited by the estrogen receptor blocker ICI 182780 [33].

### AQP 3

Different kinds of human epithelia express AQP3 in their basolateral plasma membranes. In the digestive system, AQP3 is expressed in gastric mucosal tissue, in the ileum, and in the distal colon, where it helps to water and glycerol transport [101, 102]. AQP3 is also present in the upper and lower airways, where it enables osmotic water transport through the epithelial cells of the airways [103, 104]. Moreover, AQP3 is expressed in the brain, breast, liver, pancreas, ovary, prostate, and bladder [102, 105, 106]. Recent reports have implicated many aquaporins in cancer, and mounting evidence shows that AQP3 is crucial for cancer development and metastasis [11, 107–109] [110].

AQP3 was reported to be highly expressed in lung-adenocarcinoma [36], Colorectal Carcinoma [37], cutaneous, renal [35], esophageal and oral SCC [38]. A greater tumour phase was related with lower levels of AQP3 expression in an investigation of 94 patients with bladder cancer [39]. Reduction of AQP3 expression was discovered to have a vital function in the course of urothelial bladder cancer and was related with a shorter progression-free survival (PFS) [111]. Rubenwolf and colleagues researched with Muscle-invasive bladder cancer and showed that high AQP3 expression was related with significantly enhanced PFS and cancer-specific survival (CSS) [112]. It has also been observed that AQP3 is highly expressed in human squamous cell carcinoma and that AQP3-deficient animals exhibit a reduction in the development of skin tumours [113–115]. The expression of AQP3 is enhanced in SCC of the skin [115]. A research of AQP3-deficient mice indicated that AQP3-deficient animals are resilient to the advancement of melanoma owing to a reduced concentration of glycerol in cells and ATP energy for biogenesis [113, 116], and that AQP3 was essential for skin tumour growth, as AQP3-deficient animals could not form skin tumours when exposed to a tumour initiator [116]. Further, Søland et al. discovered that lower AQP3 in hypoxic settings was associated with lesser invasive oral squamous cell carcinoma (OSCC) characteristics [40]. Finally, Kusayama and colleagues demonstrated that using specific siRNA can inhibit AQP3 function – implying a innovative role in the SCC-treatment [38]. In another study, AQP3 was shown to be overexpressed in non-small carcinoma (NSCLC), particularly adenocarcinomas, as well as well-differentiated bronchioloalveolar carcinomas and papillary subtypes. It is likely that AQP3 regulates the biological processes of lung carcinoma cells and is essential early in the development of lung ADCs [117]. AQP3 may have a role in lung cancer angiogenesis via the HIF- 2α-VEGF route, lung cancer cell invasion via the AKT-MMPs pathway, cellular glycerol uptake, or mitochondrial ATP production. In experimental NSCLC, the antitumour benefits of AQP3-targeting shRNA were found, as shown by the suppression of AQP3 ablation for lung cancer development and longer life in preclinical experiments [117]. In individuals with invasive breast ductal carcinoma, an increase in AQP3 expression is connected with a higher histopathological grade and lymph node metastasis [41]. In cell cultures, AQP3 expression was linked to enhanced metastasis of T47D [41], MDA-MB-231, and DU4475 [118] breast cancer cells, but AQP3 silencing drastically decreased metastasis in comparison with normal cells. Furthermore, AQP3-mediated H_2_O_2_ transportation into cells was required to control Akt phosphorylation and consequent targeted cell migration of chemokine (C-X-C motif) ligand 2 (CXCL2)-dependent breast cancer cells in vitro [118]. These findings point to AQP3 as a regulator of cell-migration. However, Overexpression of AQP3 in T47D breast cancer cells reduced E-cadherin protein levels while increasing Snail expression [41]. AQP3 has the ability to be a predictive biomarker in Breast Ductal Carcinoma and can be linked to its function in cell migration, which is promoted by channelling both water and glycerol – which results in the production of lamellipodia, facilitating cell movement and migration [41, 113].

The AQP3 gene has an estrogen-responsive component and increases its expression in response to estrogen stimuli, implying an association between AQP3 and estrogen receptor positive breast cancer. Furthermore, AQP3 knockdown prevented the metastasis of breast orthotropic xenographs to the lungs in vivo [118], backing up the function of AQP3 in cancer metastasis. Moreover, In colorectal cancer, both AQP3 and epidermal growth factor receptors have a function in tumour progression and metastasis; consequently, both may be suitable candidates for suppression [35]. The suppression of AQP3 by siRNA improved the susceptibility of prostate carcinoma cells to cryotherapy [119]. AQP inhibition had similar effect on breast cancer cell lines [120, 121]. RNAi inhibited the proliferation and intrusiveness of XWLC-05 lung tumour cells while increasing the action of matrix metalloprotease-2 (MMP2) [122]. Upregulation of AQP3 increased the development of SGC7901 and MGC803 gastric cancer cells, but inhibiting endogenous AQP3 inhibited growth [123]. In the same study, overexpression of AQP3 was observed to be linked with inhibition of E-cadherin expression and overexpression of vimentin and fibronectin expression. Nevertheless, knockdown of AQP3 in pancreatic BXPC3 and HPAFII cancer cells reduced cell growth and spread [124]. AQP3 expression is higher in stomach adenocarcinoma tissues in comparison with normal gastric mucosa [125]. Poor prognosis for gastric cancer is predicted by epithelial mesenchymal transition (EMT) related proteins controlled by AQP3. In vitro, AQP3 also endorses the proliferation, invasion, and migration of gastric cancer cells through the PI3K/AKT/SNAIL signaling pathway [123]. Moreover, Overexpression of AQP3 in gastric cancer cells promotes cisplatin resistance through autophagy, indicating that the invention of AQP3-based tumour treatments might act a significant function in the prospective remedy of gastric cancer [126]. In another investigation, Zhou et al. observed that AQP3 endorses stem-like properties of human gastric cancer cells, and Chen et al. reported that upregulation of AQP3 is associated with epithelial mesenchymal transition-related proteins which can be correlated with poor prognosis for GC [123]. Thus, AQP3 performs an essential function in gastric carcinogenesis caused by gastric intestinal metaplasia [127], and also regulate MMP’s proteins expression [123].

Both AQP3-specific medicines and EGFR pathway antagonists may prevent the migration of colon cancer cells [37]. Chen et al [128] researched on hepatocellular carcinoma and showed that AQP3 is up-regulated in HCC and promotes the growth and invasion of HCC cells. Additionally, Serna et al. [121] and later, Peng et al. [129] observed that Auphen and dbcAMP may suppress HCC growth and may be potential therapeutic targets for HCC. AQP3 serves a crucial function in cancer biology because it modifies cellular signaling; downstream protein expression patterns, promotes tumour formation, and facilitates cellular proliferation. In the initial phases of breast cancer, AQP3 expression is increased through the FGFR-PI3K or FGFR-ERK signaling pathways in reaction to fibroblast growth factor, as well as estrogen [41, 130]. Increased AQP3 expression is associated with poor post-surgery patient survival rates, implying its use as a potential biomarker [131]. Enhanced AQP3 channel levels, which mediate H2O2 transport and induce CXCL12-cell signalling and invasion, may enhance breast cancer metastasis [41]. AQP3 also promotes cell migration and infiltration in oestrogen receptor positive breast cancer by affecting the expression of markers involved in epithelial mesenchymal transition (EMT) and actin-cytoskeleton rearrangement [33]. AQP3 may have a function in encouraging glycerol transport into the cell in the mammary gland, hence boosting intracellular ATP availability and raising nutritional needs. Consequently, suppression of AQP3 may reduce cell growth [132]. In another study, the active phosphorylated proteins extracellular signal-related kinase (ERK), protein kinase B (Akt), focal adhesion kinase (FAK), nuclear factor kappa light chain enhancer of activated B cells (NF-kB), and Src protein tyrosine kinase decreased when AQP3 was experimentally knocked down in breast cancer MDA-IBC-3 cell lines [133].. Additionally, when AQP3 is inhibited, the transfer of extracellular H2O2 into cells is reduced, resulting in decreased tumour development [118]. Based on these observations, it is hypothesized that elevated intracellular H2O2 levels serve as a second messenger during epidermal growth factor receptor signaling, hence promoting tumour formation. AQP3 was identified as a crucial and required element for the FGF-2-promoted migration of human breast cancer cells [130]. FGF-2 could stimulate AQP3 production and cell motility via the FGFR-PI3K or FGFR-ERK signalling alleyways, which were inhibited by AQP3 loss. AQP3 expression in breast cancer cells was boosted by the addition of 5′-deoxy-5-fluoropyrimidine nucleosides (5′-DFUR), which was utilised in the chemotherapy of solid tumours [134]. It was suggested that AQP3 may be a factor limiting the therapeutic action of 5′-DFUR, because of AQP3-deletion lowered the drug’s effectiveness - making it a possible chemotherapeutic target in the development of a cancer therapy combination approach. However, it remains unproven if AQP3 may be employed as a breast cancer predictive biomarker or potential target. Inhibition of AQP3 reduced the intrusiveness of DU-145 and PC-3 prostate cancer cells, which was accompanied by a reduction in MMP3 mRNA levels and function. Inhibiting extracellular signal-regulated kinase (Erk) 1/2 activity similarly dampened these activities, although the machineries linking AQP3 to the Erk 1/2 pathway have yet to be identified [135]. The participation of AQP3 in enhanced cell mobility and metastatic spread lends credence to an EMT role. However, AQP3 mRNA was detected in both normal and malignant epithelia of human prostate tissues, but not in the mesenchyme, and its suppression improved the susceptibility of prostate cancer cells to cryotherapy [105, 119]. In one investigation, curcumin was found to downregulate AQP3 expression and to reduce cell migration in CaOV_3_, a cell line of human ovarian cancer [136].

### AQP 4

Aquaporin-4 (AQP4) governs brain water homeostasis and is the most prevalent water channel in the brain, spinal cord, and optic nerve. In the central nervous system, AQP4 is prevalently found in astrocytes and ependymal cells lining the ventricles, with the maximum expression on perivascular astrocytes end feet that enclose blood vessels. The AQP4 concentration is highest in the area of the astrocyte nearest to the blood artery [137, 138]. In edematous astrocytomas and metastatic tumours, AQP4 expression is upregulated [42]. Utilising D54MG glioma cells transfected with AQP4; McCoy et al. revealed that protein kinase C (PKC) action affects water penetrability via AQP4 phosphorylation. PKC excitation with phorbol 12-myristate 13-acetate or thrombin increased AQP4 phosphorylation, decreased water penetrability, and dramatically hindered cancer cell migration. Chelerythrine’s suppression of PKC decreased AQP4 phosphorylation while increasing water porousness and tumour cell migration [139]. Mou et al. [43] found that AQP4 expression was greater in the tumour and maximum in the peritumoral tissue in gliomas. Furthermore, AQP4 protein expression in tumour tissue of gliomas of various degrees was not statistically unusual, and the grade of peritumoral oedema positively linked with AQP4 protein expression, which in turn connected with VEGF and hypoxia inducible factor 1 alpha (HIF-1) expression. Increased expression of AQP4 in CNS tumours was related to considerable peritumoral oedema, as shown by Ng et al. The ablation of AQP4 in astroglial cells greatly inhibited cell motility more towards a stab injury in adult mouse brain, and glial wound development was disrupted in AQP4-null animals, with decreased migration of reactive astroglia toward a spot of brain damage [13, 140]. AQP4 may have a role in the migration and invasion of brain tumours, and it may fast-track tumour migration by enabling the quick alterations in cell volume that occur when cell shape alters. Noel et al. [141] investigated changes in the expression and localization of AQP4, dystroglycan, agrin, and MMP-2, MMP-3, and MMP-9 in human primary brain tumours using freeze-fracture electron microscopy, immunohistochemistry, and Western blotting. They disclosed that AQP4-expression was greater in glioma than in normal tissue, and that increase in MMPs immunoreactivity was related to the loss of agrin and dystroglycan, correspondingly. Cell movement, actin polymerization, and apoptosis are impaired by AQP4 genetic depletion [142, 143]. In addition, AQP4 expression is higher in malignancies of the brain, lung, and thyroid [35]. Furthermore, AQP4 deletion obstructed glioma cell motility and F-actin polymerization, both of which are essential in cell-cell adhesion [142]. However, it has been proposed that AQP4 regulates tumour cell migration and invasion through cytoskeleton reorganization and cell adhesion modulation [144]. Nonetheless, AQP4 was also identified as an anti-death target for glioblastoma treatment, as indicated by the discovery that siRNA-facilitated AQP4 repression caused brain tumour cell death [143]. The AQP4-IgG found in individuals with NMO is uniquely coupled to AQP4 and decreases the water penetrability of AQP4, resulting in complement-dependent cytotoxicity in astrocytes [145]. Now, the monoclonal antibody precisely for AQP4 has been designed [146]. Therefore, it has been hypothesized that AQP4-specific antibody coupled to a toxin might be employed to specifically harm AQP4-expressing glioblastoma cells [11]. The levels of AQP4 protein and mRNA expression in GC tissue were considerably lesser than in normal tissue [147]. In addition to human thyroid cancer cell lines, the expression of AQP4 in normal, hyperplastic, and neoplastic thyroid tissues was varied – found in 100% of follicular adenomas, 90% of follicular carcinomas, and 85% of papillary carcinomas; while lacking in all medullary carcinomas and undistinguishable carcinomas [148].

The fact that AQPs can be a valid therapeutic target was demonstrated by Kitchen et al. in 2020 [2]. The study reveals that CNS edema is linked to both heightened overall AQP4 expression and the movement of AQP4 within cells towards the blood-spinal-cord barrier (BSCB). When AQP4 translocation to the BSCB is hindered using pharmacological methods, the onset of CNS edema is prevented, and the recovery of function is encouraged in rats with injuries [149]. This role has recently been affirmed by Sylvain et al. in their work published in BBA 2021. They effectively showcased that targeting AQP4 leads to a substantial reduction in cerebral edema during the initial acute phase of stroke, using a photothrombotic stroke model. Additionally, they established a connection to brain energy metabolism through the observed increase in glycogen levels [150].

Nito et al. [151] also made an interesting study that suggests the regulation of AQP4 expression by MAPK/JNK pathway post-ischemic injury, that leads to the formation of brain edema [151]. It was observed that a selective inhibitor of p38 MAPK or that of JNK performed significant attenuation of the return of AQP4 to its homeostatic level. This led to a significant decrease in cell death.

### AQP 5 & 6

The submandibular gland of rats served as the original source for the cloning of aquaporin-5 (AQP5), which has now been found to be widely distributed in the human body. Specifically, it manifests in the gastrointestinal, renal, pulmonary, integumentary, and genitourinary systems in addition to the sense organs. Being largely selective for water, AQP5 is essential for the regulation of water flow in many physiological systems.

By regulating tumour growth and apoptosis, AQP5 can play a significant role in carcinogenesis and tumour progression. AQP5 was observed to be not only connected with breast cancer cell proliferation, but also with cell migration [44]. Additionally, there were differences in the AQP5 expression profiles between benign tumours and invasive ductal carcinoma, with AQP5 expression in ductal epithelial cells’ apical domains compared to overexpression in cancer cells with duct loss and loss of apical polarity, implying a contribution to the progression of breast cancer. In the MCF7 breast cancer cell line, knocking down AQP5 inhibited cell migration and proliferation, showing that this isoform is critical in tumour dissemination. There was a favourable correlation between AQP5 and Ki-67 expression levels and involvement of lymph nodes in cervical cancer, which was strongly correlated with AQP5 and Ki-67 [45]. Through the phosphorylation of the PKA consensus domain in its cytoplasmic loop D, AQP5 has been shown to be capable of directly stimulating cell proliferation by triggering the Ras cascade pathway [152]. AQP5 expression has also been observed to be elevated in cervical [45] and endometrial cancer [46]. Inhibiting AQP5 reduced the migration and proliferation of 3AO ovarian carcinoma cells along with the rates of tumour development, suggesting that AQP5 is engaged in the genesis and dissemination of tumours [153]. According to numerous research, AQP5 expression levels are directly correlated with tumour stage, lymph node metastases, and poor diagnosis, suggesting that AQP5 may be used as a potential biomarker for ovarian cancer [154]. Increased expression of AQP5 in prostate cancer was linked to the stage of the tumour node metastasis and lymph node metastasis [47]. In an experiment, squamous cell carcinoma (SCC) of the tongue has been shown to have an increased expression of AQP5 [155]. AQP5 suppression was also found to impede cell proliferation in a tongue SCC cell line, most likely through distortion of actin alignment and decrease of integrin a5 and b1nian expression in the early phase; suppressing MAPK signalling cascade in the second stage. Yang and colleagues demonstrated that through inhibiting the EGFR/ERK/p38 MAPK signalling pathway, AQP5 gene silencing suppressed the proliferation, inhibited the migration, and increased the apoptosis ratios of human glioma cells [48].. Comparing CML cell populations and myeloid specimens to lymphocytes from peripheral blood and healthy bone marrow cells, Chae and colleagues revealed that AQP5 is upregulated. In addition, they established a connecting link between enhanced AQP5 activity and a rapid or explosive crisis phase. There are indications suggesting AQP5 upregulation is linked with increased cellular proliferation, which relates closely to heightened BCR-ABL1 phosphorylation at Tyr177 and Akt phosphorylation at Thr308, correspondingly. AQP5 ablation inhibits these two critical cells signalling compounds for CML cell growth, as demonstrated by siRNA studies. It has also been demonstrated that inhibiting AQP5 induces caspase 9 operations, which results in a rise in the quantity of cells experiencing apoptosis [156]. Increased AQP5 activity was also associated with susceptibility to imatinib mesylate, a tyrosine-kinase antagonist utilised to treat chronic CML. Jung et al. [44] found that inhibiting AQP5 or triggering hyperosmotic stress in MCF-7 cells diminishes AQP5 activity and has a detrimental effect on cell growth and migration. The proliferation capacity of lung cancer cells was shown to be favourably linked with the amount of AQP5 expression [49]. Patients with lung cancer who expressed AQP5 significantly had a greater rate of tumour recurrence and a shortened tumour-free survival rate [157]. Nevertheless, Machida and colleagues. Discovered that AQP5 activity was favourably linked with the level of cell proliferation and differentiation but not to prognosis [158].

In NSCLC tissues, AQP5 has multiple associations with the progression of cancer. AQP5 is expressed at considerably higher amounts in adenocarcinomas than in cell carcinomas. Tissues with lymph node metastases expressed AQP5 at elevated amounts than tissues without it. AQP5 correlated positively with the tumour-node-metastasis stage of NSCLC [157]. Overexpression of AQP5 may induce lung cancer cell spread and invasion by triggering the EGFR/ERK/p38 MAPK cascade [49]. AQP5 small interfering RNA (siRNA) treated LAMA84 CML cells showed a significant decrease in cellular proliferation when compared to control siRNA administered cells, according to a study on human chronic myelogenous leukaemia (CML) [156]. Zhang and colleagues found that lung cancer cells with elevated AQP5 activity had a greater proliferative capacity than those with low AQP5 activity [49]. Chen and colleagues utilised short hairpin RNA to inhibit the translation of the AQP5 gene; thus, the migration ability of lung cancer SPCA1 cells was significantly diminished [159]. Kang and colleagues. Observed the upregulation of AQP5 enhanced phosphorylation of the protein retinoblastoma (Rb) in colorectal cancer, which could be achieved via stimulating the Ras/ERK/Rb signalling cascade [50]. AQP5 upregulation is also associated with lymph node metastases and lymphovascular infiltration, which are all connected with the severity of gastric carcinoma [51, 52]. In HT-29 cell lines, AQP5 suppression revealed that AQP5 activity stimulates cell growth even in the context of chemotherapeutic intervention. In fact, it was observed that AQP5 expression is positively linked with the activity of multidrug resistance (MDR) molecules like P-glycoprotein [160]. In another experiment it was observed that AQP5 siRNA or a p38 MAPK antagonist inhibited p38 phosphorylation in HT-29 cells, suggesting this cascade pathway is involved in MDR colon cancer [160]. AQP5 has been demonstrated to engage with the Ras cascade in colon cancer, suggesting that these might play a greater role in communication than previously thought. Upregulation of AQP5 in lung adenocarcinoma and colorectal cancer has been identified, making it a prognostic biomarker. AQP5 was discovered to be highly elevated in 14 of 45 colorectal cancer tumour samples, moderately elevated in 29 of them, and not detectable at all in two of them. Elevated expression levels were linked with TNM stage (the categorization of cancerous tumours), distant metastasis and lymph node metastasis suggesting that AQP5 level of expression could be employed as a predictive biomarker. In addition, those without AQP5 activity had a greater cumulative life expectancy [161]. AQP5 is also postulated as a predictive biomarker for colorectal carcinoma, with AQP5 concentrations correlated with the number of cancerous cells in circulation and the likelihood of liver metastases [50]. In colon carcinoma cells, genetic silencing of AQP5 increased chemosensitivity and inhibited p38 MAPK pathway [160, 162]. Cairicoside E (CE), a glycoside produced from the resin of *Ipomoea cairica* (Convolvulaceae), inhibited migration of colon cancer cells via inhibiting EMT. Interestingly, in the absence of AQP5, CE seemed to have no impact on EMT, showing that the inhibitory action of CE on EMT is dependent on the suppression of AQP5. Furthermore, it was discovered that transforming growth factor 1 (TGF 1) induced AQP5 translation, and that AQP5 upregulation elevated p Smad2/3, hence triggering EMT [163]. In colon carcinoma cells, ablation of AQP5 may increase chemosensitivity by inhibiting the p38 MAPK signalling pathway [160].

Huang and colleagues revealed that AQP5 expression was significantly elevated in gastric cancer tissues and that suppressing AQP5 expression with acetazolamide inhibited the proliferation and invasiveness of gastric cancer cells [51]. HgCl2, an AQP inhibitor, markedly reduced the number of differentiated cells and alkaline phosphatase activity in MKN45 gastric carcinoma cells that firmly showed AQP5 activity [164]. By using siRNA to inhibit AQP5 activity in HT 29 colon carcinoma cell lines, the susceptibility of these populations to the chemotherapeutic agents cisplatin (DDP) and 5 fluorouracil (5 FU) was enhanced. F furthermore, impedance to imatinib mesylate, a tyrosine kinase inhibitor used in the chronic stage of CML intervention, was associated with increased AQP5 expression. AQP5-targeting siRNA decreased the rate of cell proliferation in CML cells [156, 160]. Rodrigues et al. [165] established that AQP5 possesses a very effective peroxiporin function, with external oxidative stress stimuli restoring the inhibition of cancer cells’ ability to migrate caused by AQP5 knockdown. As a result, they emphasised the importance of AQP5 in dynamic fine-tuning of intracellular H2O2 levels [165], which is critical for redox signalling and cell fate control [166]. Hence, AQP5 may have potential in anticancer treatment. Likewise, the identification of three AQP5 mediating miRNAs (miR-19a-3p, miR-1226–3p, and miR-19b-3p) which reduce breast cancer cell motility by lowering AQP5 translation supports the concept of additional research to prove it as potential therapeutic target [167]. In breast cancer cell lines, AQP5 knockdown increased the MAPK signal transduction pathway, decreasing cell invasion and metastasis and growth while enhancing chemosensitivity, indicating that AQP5 could be advantageous as a predictive biomarker and therapeutic target [168, 169]. Tumours that show elevated expression AQP5 also have a phosphorylated cAMP-protein kinase (PKA) consensus domain, which stimulates cellular proliferation. Targeting Ser156 in AQP5 is a promising therapeutic strategy due to its role in lung cancer cell growth and invasion [170]. Conclusion of another experiment revealed that it is possible that the increased metastatic potential of lung cancer is caused, at least in part, by the fact that AQP5-positive cells lose epithelial cell markers and hasten EMT by stimulating c-Src through the SH3 binding domain [49]. The preferential phosphorylation of the cAMP-protein kinase (PKA) consensus site on AQP5 promoted cancer cell proliferation. By phosphorylating Ser156 at the PKA consensus site, Ser156 mutations in lung cancer cells have been demonstrated to play a significant role in cancer proliferation and invasion [171]. As a result, Ser156 in AQP5 presented a novel therapeutic target.

It has been shown that AQP5 induces tumour growth via stimulating the Ras-MAPK cascade, cyclin D1/CDK4 complexes, phosphorylating retinoblastoma protein in the nucleus, and culminating in the expression of cell proliferation-related genes [50]. Inhibitors of AQPs derived from heavy metals like silver, gold, or ruthenium were also studied for their potential as anticancer therapies [172, 173]. Cisplatin, a popular chemotherapy treatment, suppressed the production of AQP5 that is overexpressed in ovarian tumours and correlated with ascites and lymph node metastasis [173]. Inhibition of AQPs by HgCl2 dramatically reduced the percentage of differentiated cells and alkaline phosphatase activity in the human gastric cancer cell line MKN45 that stably expressed AQP5 [164]. Yang and colleagues demonstrated that AQP5 protein had a significant relationship with cell proliferation rate, and also that cisplatin may elicit a concentration-dependent reduction in AQP5 activity in human ovarian cancer CAOV3 cells [174]. Epigallocatechin gallate suppressed ovarian cancer SKOV3 cell growth and induced apoptosis in a time and dose dependent manner by suppressing AQP5, which could be associated with nuclear transcription factor, nuclear factor kappa B (NF-kB) [175]. Kudou et al. also found that AQP5 inhibition impedes HCC metastasis and EMT via deactivation of the NF-кB signaling pathway [176], while Yang et al. observed that Topotecan negatively regulates AQP5 and NF-kB activity [154]. Although the critical role of AQP6 wasn’t associated in different cancers its expression was down regulated in ovarian cancer [177].

### AQP 7, AQP 8 & AQP 9

**Fig. 2:**
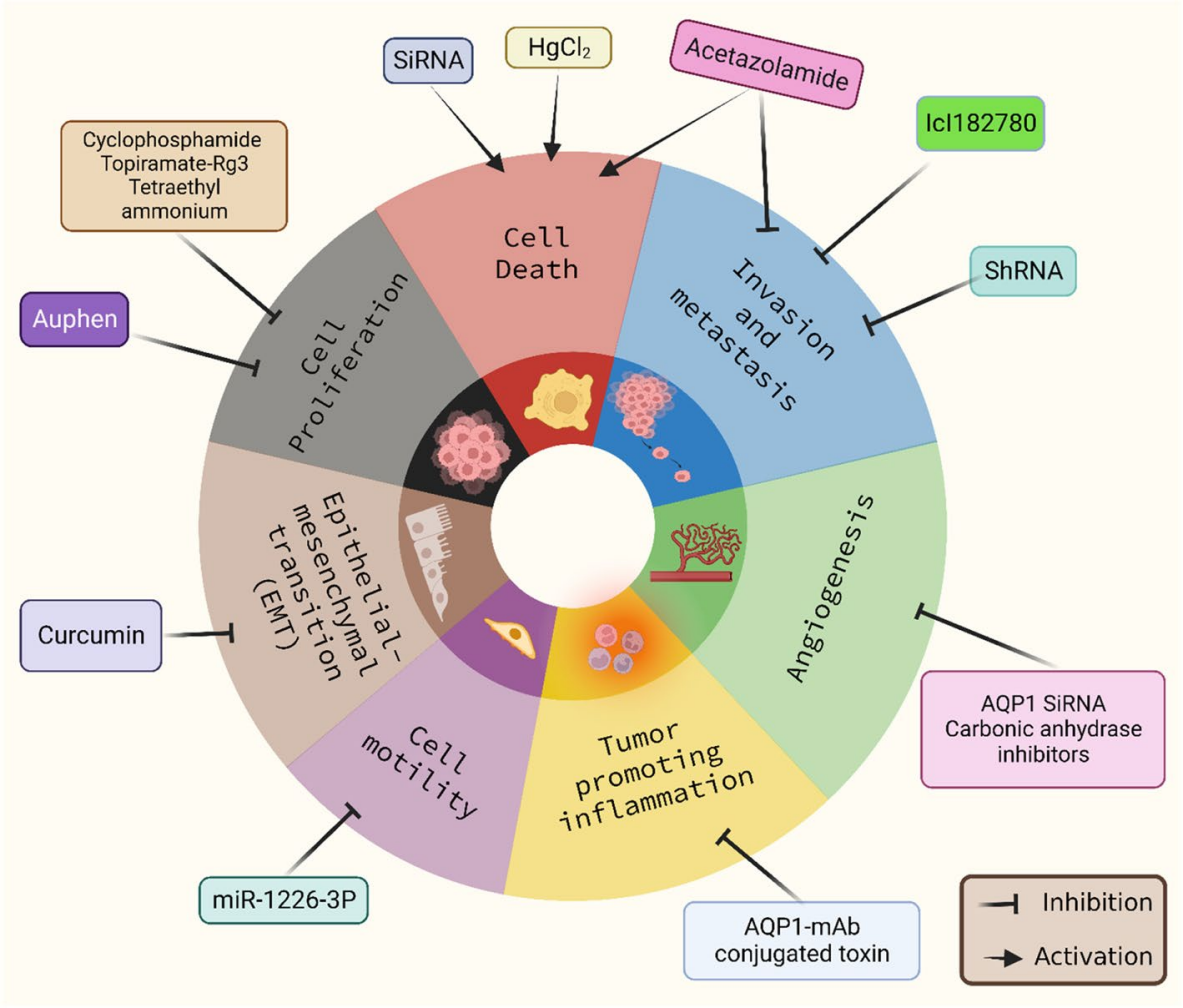
AQP inhibitors targeting different hallmarks of Cancer. CA inhibitors, AQP1 -mAb conjugated toxin, miR-1226-3P, and Auphen targets distinct hallmarks of cancer such as angiogenesis, inflammation, cell motility, and cell proliferation respectively whereas Curcumin, HgCl2, acetazolamide, ICL182780 inhibits EMT, activates growth suppressors, triggers cell death, and inhibits invasion and metastasis respectively

Thyroid malignancies had higher levels of AQP7 expression, while brain or ovarian cancers have elevated amounts of AQP9 [53]. AQP8 expression was related to the extent of invasion of cervical cancer cells and was appears to be strongly connected with ERK1/2 activity in cervical carcinoma. In ovarian cancer, AQP 9 was shown to be downregulated whereas AQP 8 stayed constant, however their functions are unknown [25, 177]. AQP 8 has the power to influence NOX-derived H2O2 transport across the plasma membrane, hence modulating redox signalling associated with acute leukaemia cell growth [54]. Foss et al. addressed the expression and localisation of AQP1 and AQP 9 in glioblastoma biopsies, malignant stem cells developed in culture as neurospheres, and differentiated cells differentiated from these malignancies Fig. 2.

They showed a significant increase in AQP9 mRNA expression in tumour stem cells, followed by high expression in differentiated cells, indicating that AQP9 may play a key role in glioblastoma carcinogenesis [55]. The increased arsenic uptake observed by Miao and colleagues suggests a potential role for increased AQP9 expression in the evolution of arsenic tolerance in human lung carcinoma cells [56]. In the first study of its kind, Chang and colleagues, found that human oesophageal cancerous cells produced AQP8 and that epidermal growth factor increased AQP8 translation and cell motility in these cells via the EGFR/ERK1/2 signalling pathway [57]. Nevertheless, AQP8 expression is diminished in hepatocellular and colorectal malignancies [178]. Because of its reduced expression, which minimizes apoptotic stimulation, AQP9 is a potential therapeutic target for HCC [35]. In hepatocellular carcinoma, decreased AQP9 expressions may suggest resistance to apoptotic stimuli. AQP9 expression was negatively mediated in HCCs and was mostly found in non-tumorigenic liver tissue [179] Reduced AQP9 expression in HCCs can improve the sensitivity to apoptotic stimuli in hepatocellular carcinoma cells. It was demonstrated that AQP9 may be an unique therapeutic or diagnostic target for hepatocellular cancer [180].

Recently AQPs in tumour biology are starting to play several functions, the molecular pathways required for AQP-mediated cell growth and ability to migrate must be better defined. We have summarized the investigations involving various types of AQPs with the respective inhibitory molecules for specific Cancer cell lines in Table 2. However, it is unknown if the ability of AQPs to transport water is relevant or if there are correlations between AQPs and oncogenes that have not yet been recognised. In addition, further research is required to develop non-toxic AQP antagonists that might be used to characterise AQP roles and as new cancer treatments. AQP antagonists, which may target tumour invasion, dissemination, and angiogenesis, could be combined with existing cancer treatments targeting tumour cell proliferation.

**Table 2:** Investigations involving Types of AQPs with the respective inhibitory molecules for specific Cancer cell lines

AQPs	Inhibitory molecule	Type of Study/Studied on	Inhibited Process/Outcome	Reference
AQP1	Ion channel Blocker	H2T9	Cell Migration	[60]
	Carbonic Anhydrase inhibitor-Acetazolamide	Glioma cell line	Invasion and angiogenesis	[81, 87]
	AQB050	primary malignant mesothelioma cells	Reduction in cell growth	[89]
	cyclophosphamide, topiramate and anaesthetic drugs		AQP1 inhibitors	[181]
	Rg3	PC-3 M prostate cancer cell line	Inhibited p38 MAPK pathway	[92]
	Tetraethyl ammonium	J82 human bladder cancer cells	Blocks AQP1	[95]
	microRNA-320 AQP1 siRNA	LTEP-A2 and LLC lung cancer cell lines; HMEC-1 human endothelial cells	Downregulating matrix metalloprotease; Lack of F-actin polarisation	[68, 79, 80]
	shRNA	U2OS and MG63 osteosarcoma	Decrease in proliferation, Reduced TGF-B signalling	[31]
AQP2	Oestrogen receptor blocker ICI182780	Ishikawa cells in human Endometrial cancer	Reduced invasion, proliferation and migration	[33]
AQP3	RNAi	XWLC-05 Lung cancer cells Pancreatic BXPC3, HPAFII MDA-IBC3 breast cancer cell lines	Reduced proliferation and invasion Reduced cell growth and proliferation Diminished tumour migration	[122, 124, 133]
	Auphen and dbcAMP	DU-135 & PC-3 prostate cancer cells HCC cells	Reduced matrixmetalloprotease3 Reduced HCC development	[129]
	Curcumin	CaOV3 ovarian cell line	Reduced cell migration	[132]
AQP4	siRNA mediated knockdown; Monoclonal AQP4-IgG conjugated with toxin	Glioblastoma cells	Cellular apoptosis and Selective cytotoxicity respectively	[11, 142, 143]
AQP5	RNAi	MCF7 Breast cancer cell line Tounge SCC cell line LAMA84 CML cells	Reduced cell migration & proliferation Decreased cell migration Reduced cell proliferation	[45, 48, 156]
	Short hairpin RNA	Lung adenocarcinoma SPCA1 cell line	Reduced cell migration	[159]
	P38 MAPK inhibitor	HT-29 cells colon cancer	Reduced p38 phosphorylation	[160]
	Cairicoside E	Colon cancer cells	Decreased EMT	[163]
	Acetazolamide	Gastric cancer cells	Reduced proliferation and invasiveness	[51, 52]
	HgCl_2_	MKN45 gastric cancer cells	Reduced number of differentiated cells	[164]
	miR-1226–3p, miR-19a-3p, and miR-19b-3p	Breast cancer cells	Reduced cell motility	[167]
	Epigallectocatechin gallate	SKOV3 ovarian cancer cells	Promoted apoptosis and inhibited cell proliferation	[175]

## Conclusion and future perspectives

AQPs have been connected with tumour growth, angiogenesis, metastasis, tumour cell adhesion, and tumour related edema in several malignancies. Therefore, it would be beneficial for us to utilise AQPs as prospective cancer treatment. Now, AQP-specific monoclonal antibodies, small-molecule inhibitors of AQP synthesis including AQP-induced water penetration, and heavy-metal-reactive cysteine-based antagonists have all been developed and shown to be effective at inhibiting AQPs. Eleven patients who had their parotid glands irradiated due to head or neck cancer radiation treatment participated in the Phase I clinical trial using AQP1-cDNA transfer therapy [182]. It is extremely inspiring that AQP gene transfer has been developed and implemented in clinical therapy. But the effectiveness and safety require additional study.

Recent trends in the field of targeting the molecular and signalling mechanisms of AQP4 and astrocytes have shifted towards more nuanced and sophisticated approaches, departing from traditional methods [183]. We would like to discuss the following emerging trends: Selective Modulation: Instead of simply inhibiting AQP4 activity, researchers are now exploring ways to selectively modulate its function. This involves influencing the specific roles of AQP4 in various cellular processes without completely blocking its activity [183, 184]. Astrocyte-AQP4 Interactions: Focus has increased on understanding the complex interactions between AQP4 and astrocytes. Researchers are investigating how AQP4 influences astrocyte function and vice versa, aiming to identify ways to manipulate these interactions for therapeutic purposes [185]. Downstream Signaling Pathways: Rather than concentrating solely on AQP4 itself, scientists are delving into the downstream signaling pathways triggered by AQP4 activation [186]. Targeting these pathways offers a broader perspective on the role of AQP4 and potentially uncovers new intervention points. Biased Ligands and Allosteric Modulators: Novel drug design strategies involve creating biased ligands or allosteric modulators that can subtly alter AQP4 function. These compounds can produce specific effects in certain cellular contexts, leading to more precise therapeutic outcomes [186]. Gene Editing Techniques: With advancements in gene editing technologies like CRISPR-Cas9, researchers are exploring ways to modify AQP4 and astrocyte genes to achieve desired effects. This opens avenues for directly altering cellular behavior in a targeted manner. Nanotechnology and Drug Delivery: Innovative drug delivery methods, such as nanotechnology-based approaches, are being developed to precisely target AQP4 and astrocytes. These methods aim to enhance drug efficacy and minimize off-target effects [187]. Functional Blockade: Beyond traditional pore-blocking, newer strategies involve functionally blocking AQP4 in specific cellular compartments or under certain conditions. This approach aims to maintain AQP4’s beneficial functions while preventing detrimental effects. Combined Therapies: Combining interventions targeting AQP4 and astrocytes with other therapeutic strategies, such as anti-inflammatory drugs or neuroprotective agents, is gaining attention. This multi-pronged approach addresses the complexity of CNS disorders more comprehensively. Systems Biology Approaches: Systems biology techniques, like computational modeling and network analysis, are aiding researchers in unraveling the intricate interactions between AQP4, astrocytes, and other molecules. This holistic perspective guides the development of targeted interventions [183, 188]. In conclusion, the recent trends in targeting AQP4 and astrocytes involve a shift towards more precise, context-specific, and multifaceted approaches. These innovative strategies aim to harness the therapeutic potential of AQP4 and astrocyte modulation while minimizing unwanted side effects.

AQPs-specific monoclonal antibodies, AQPs-targeted inhibitors, and AQP gene transfer are all prospective treatments involving AQPs based on the outcomes of current investigations. To comprehend the relevance of AQP to homeostasis and cellular environment, it is important to understand AQP’s role and gating features. This is therapeutically relevant since altering AQP activity may have therapeutic benefits. Moreover, the importance of aquaglyceroporin-mediated glycerol trafficking in cell growth and adipocyte metabolic activity, as well as its involvement in the genesis of cancer, indicate the aquaglyceroporin subfamily as a potential therapeutic target [35].

However, the success rate for the development of small-molecule AQP inhibitors seems to be substantially lower than that of other membrane proteins like solute transporters, ion channels, and membrane receptors. The small size of the functional AQP monomer and its extremely narrow pore diameter, hinders the entry of small particles, contributing to its unfortunate druggability. Since AQPs are simple, passive pores, they are short of specialized gating and transport systems that can be regulated by microscopic particles. Moreover, electrostatic interactions between AQPs are confined to those involving hydrogen-bond donors and acceptors, as opposed to interactions involving charges, due to the neutral nature of the AQPs’ molecules. Finally, alterations in the extrinsic or intracellular areas of AQPs do not affect water permeability across the channel, suggesting that the attachment of an antagonist should happen deep within the tiny AQP channel to physiologically hinder water transmission.

Although, recent research showed that AQPs play several functions in tumour development, the molecular pathways required for AQP-mediated cell growth and ability to migrate remains to be better defined. It is unknown if the ability of AQPs to transport water is relevant or if there are correlations between AQPs and oncogenes that have not yet been recognised. Moreover, the use of humanized self-organized models, organoids, 3D cultures, and human microvessel-on-a-chip platforms, particularly those that are compatible with advanced imaging techniques like TEM and expansion microscopy because they allow real-time monitoring of changes in AQP dynamics, may be able to open up new windows of opportunities to study Aquaporin and develop better treatments. In addition, additional investigation is essential to develop non-toxic AQP antagonists that might be utilised to characterise AQP roles and as new cancer treatments. AQP antagonists, which may target tumour invasion, dissemination, and angiogenesis, could be combined with existing cancer treatments targeting tumour cell proliferation.

While AQPs have been confirmed as significant drug targets, there has not been a single approved drug that effectively addresses them. This underscores the need for studies akin to the present one, which offer different avenues for targeting AQP function beyond the conventional approach of blocking their pores. Currently, AQPs are not amenable to drug development. However, there have been notable strides in utilizing high-throughput screening and computer-aided drug design. Aldewachi et al. (2021) comprehensively reviewed these advancements, which offer a fresh perspective that could facilitate the validation of AQP targets in forthcoming research endeavors [189].

## Data Availability

All data are incorporated into the article.

## Abbreviations

5′-DFUR: 5′-deoxy-5-fluoropyrimidine nucleosides
AQP: Aquaporin
ADC: Antibody-drug conjugates
CA II: Carbonic anhydrase 2
CC: Cholangiocarcinoma
CSS: Cancer specific survival
CML: Chronic myelogenous leukaemia
EGFR: Epidermal growth factor receptor
EMT: Epithelial mesenchymal transition
ERK: Extracellular signal-related kinase
FAK: Focal adhesion kinase
FGF-2: Fibroblast growth factor-2
HCC: Hepatocellular carcinoma
HIF-1: Hypoxia inducible factor 1 alpha
IHC: Immunohistochemistry
JNK: C-jun N-terminal kinase
LLC: Lewis lung carcinoma
MDR: Multidrug resistance
MEKK1: Mitogen activated protein kinase kinase
MIP: Major Intrinsic Protein
MMP: Matrix metalloproteinases
MMC: Mitomycin C
mRNA: Messenger RNA
NF-kB: Nuclear factor kappa B
NSCLC: Non-small cell lung cancer
OSCC: Oral squamous cell carcinoma
PAK: p21-activated kinase
PFS: Progression-free survival
PKC: Protein kinase C
Ras: Rat sarcoma virus
Raf: Rapidly accelerated fibrosarcoma
RhoA: Ras homologue family member A
ROS: Reactive oxygen species
SCC: Squamous cell carcinoma
TEAþ: Tetraethyl ammonium
TNM Stage: Tumour (T), Nodes (N), and Metastases (M)
